# Glucose Control, Disease Burden, and Educational Gaps in People With Type 1 Diabetes: Exploratory Study of an Integrated Mobile Diabetes App

**DOI:** 10.2196/diabetes.9531

**Published:** 2018-11-23

**Authors:** Cornelis J Tack, Gerardus J Lancee, Barend Heeren, Lucien JLPG Engelen, Sandra Hendriks, Lisa Zimmerman, Daniele De Massari, Marleen MHJ van Gelder, Tom H van de Belt

**Affiliations:** 1 Department of Internal Medicine Radboud University Medical Center Nijmegen Netherlands; 2 Radboud REshape Innovation Center Radboud University Medical Center Nijmegen Netherlands; 3 Royal Philips Eindhoven Netherlands; 4 Department for Health Evidence Radboud Institute for Health Sciences Radboud University Medical Center Nijmegen Netherlands

**Keywords:** diabetes, app, self-care, medication suggestion, disease management, diabetes mellitus, type 1, mobile applications

## Abstract

**Background:**

Self-monitoring and self-management, crucial for optimal glucose control in type 1 diabetes, requires many disease-related decisions per day and imposes a substantial disease burden on people with diabetes. Innovative technologies that integrate relevant measurements may offer solutions that support self-management, decrease disease burden, and benefit diabetes control.

**Objective:**

The objective of our study was to evaluate a prototype integrated mobile phone diabetes app in people with type 1 diabetes.

**Methods:**

In this exploratory study, we developed an app that contained cloud-stored log functions for glucose, carbohydrates (including a library), insulin, planned exercise, and mood, combined with a bolus calculator and communication functions. Adults with diabetes tested the app for 6 weeks. We assessed the feasibility of app use, user experiences, perceived disease burden (through questionnaires), insulin dose and basal to bolus ratio, mean glucose level, hemoglobin A_1c_, and number of hypoglycemic events.

**Results:**

A total of 19 participants completed the study, resulting in 5782 data entries. The most frequently used feature was logging blood glucose, insulin, and carbohydrates. Mean diabetes-related emotional problems (measured with the Problem Areas in Diabetes scale) scores decreased from 14.4 (SD 10.0) to 12.2 (SD 10.3; *P*=.04), and glucose control improved, with hemoglobin A_1c_ decreasing from 7.9% (mean 62.3, SD 8 mmol/mol) to 7.6% (mean 59.8, SD 7 mmol/mol; *P*=.047). The incidence of hypoglycemic events did not change. Participants were generally positive about the app, rating it as “refreshing,” and as providing structure by reinforcing insulin-dosing principles. The app revealed substantial knowledge gaps. Logged data enabled additional detailed analyses.

**Conclusions:**

An integrated mobile diabetes app has the potential to improve diabetes self-management and provide tailored educational support, which may decrease disease burden and benefit diabetes control.

## Introduction

### Optimizing Self-Management of Type 1 Diabetes

Type 1 diabetes is an autoimmune disease that occurs in genetically susceptible individuals and leads to the complete absence of insulin production by pancreatic beta cells [1]. It often debuts in childhood or early adolescence and requires insulin replacement therapy. To reduce the risk of long-term complications, people with diabetes aim for optimal blood glucose control, which requires self-monitoring of blood glucose levels at least four times daily, injection of rapid-acting insulin before every meal and of long-acting insulin before night (or by continuous subcutaneous insulin infusion by insulin pump), and adjustment of insulin dose based on food (carbohydrate) intake, actual glucose levels, intended physical activity, and experience (self-management) [2]. Optimal self-monitoring and self-management requires not only extensive education but also substantial efforts from people with diabetes. Still, and frustratingly, episodes of high and low glucose levels are often a fact of everyday life [3]. It has been estimated that life with type 1 diabetes requires an astonishing number of health-related decisions, even estimated at about 180 per day [4]. Altogether, the self-management of type 1 diabetes presents a significant burden [5].

Technological tools and mobile apps could support people with diabetes in everyday diabetes self-management. These include systems that facilitate data logging to integrate the various relevant measurements, provide educational information, and provide decision support software. An example of decision support software is the Bolus Wizard or bolus calculator, which advises people with diabetes on meal insulin dose [6]. Although technology and innovation have the potential of making a meaningful impact on diabetes care and could offer important solutions, clinical effects of digital health care solutions are often poorly investigated [7,8], particularly regarding diabetes type 1 [9-11]. Moreover, past studies focused on a limited number of outcomes [12].

### Objectives

In close collaboration with people with diabetes, the Radboud University Medical Center (Radboudumc; Nijmegen, the Netherlands), Royal Philips (Eindhoven, the Netherlands), and Salesforce (San Francisco, CA, USA) developed a prototype of an integrated mobile diabetes app to be used on a mobile phone, including a bolus calculation function, data logging, a forum, and direct messaging with health care providers. We studied its potential effect on disease burden and assessed its feasibility and participants’ experiences with this prototype app.

## Methods

### Design and Setting

In this exploratory study, conducted at the Radboudumc in Nijmegen, the Netherlands, among adults with type 1 diabetes, we compared baseline measurements with measurements taken during and after the intervention. We also applied qualitative methods to assess users’ experiences, barriers and facilitators for using the app, and perceived effects. Finally, we analyzed objective and subjective data.

### The Mobile App

The integrated mobile phone diabetes app is a prototype diabetes mobile support software app for iOS (Apple) and Android (Google). This app was developed for use by people with type 1 diabetes and contains the functionalities presented below. These functionalities were chosen based on technical possibilities, clinical expert input, and opinions expressed by people with diabetes (n=10) who were interviewed and reviewed layout and features on two occasions during the development process. These persons were not included in this study.

- Logbook capabilities to capture key measurements: users can manually enter blood glucose levels, as well as hypoglycemic events, carbohydrate intake, injected insulin dose, expected physical activity, stress, and mood. These data can be displayed in the app as last-entered values and trend graphics (history values) of blood glucose (day, week, and month views).
- Carbohydrate intake data entry support: the “meal picker” provides a means to define personal standard meals and to look up carbohydrate contents of frequently used ingredients.
- Custom settings: users (or their health care providers) can set a target blood glucose level, alarms as reminders, and settings for the bolus calculator (eg, ratios), as well as an on-off switch for a warning if blood glucose value entries exceed individual limits. In the case of the entry of blood glucose levels above 25 mmol/L, a text message appears and direct contact with a nurse (by telephone) is offered.
- Insulin bolus advice: based on data entered and personal settings, such as the personal carbohydrate to insulin ratio, the app calculates bolus advice using a modified version of an equation by Schmidt et al [6]. We removed the insulin on board variable for safety reasons, with no dose advice given within 3 hours.
- Secure communication: people with diabetes can communicate with health care providers through a secure connection.
- Online community: people with diabetes can connect with their peers.
- Privacy and security measures: the app was developed respecting international privacy and security standards, including a secured connection. All data were coded, and all users used a fake username and credentials for the app to make sure no data were in the system that could be traced back to an individual or his or her medical data. Data were stored on a cloud server based in Europe and according to the privacy and security policy of Salesforce. Moreover, the privacy and security procedures were reviewed and approved by Radboudumc’s and Royal Philips’ privacy and security officers.

Multimedia Appendix 1 provides screenshots of the app.

### Participants

As this was an initial feasibility study, we recruited a representative sample of people with type 1 diabetes from the outpatient clinic of the Radboudumc, aged between 18 and 65 years, with a diabetes duration of at least 2 years and stable glycemic control (hemoglobin A_1c_ [HbA_1c_] between 7% and 10% [53-86 mmol/mol]), able to count their carbohydrate intake and vary their bolus insulin dose, having a body mass index between 18 and 35 kg/m^2^, and using a suitable (iOS 9 or Android 4.1. and higher) mobile phone or tablet. Participants had to be able to speak, read and understand Dutch.

Exclusion criteria were people with serious diabetes complications: severe retinopathy with poor vision (visual acuity <0.5), renal failure (glomerular filtration rate <30 mL/min/1.73 m^2^), foot amputation, recent (<6 months) myocardial infarction or stroke, any serious comorbidity deemed to significantly affect participation, a history of severe hypoglycemia (requiring third-party assistance) over the past 3 years, pregnancy or aiming for pregnancy, or total insulin need greater than 1 U/kg/day.

Eligible people with diabetes were identified by their treating physician and invited to participate by letter. Subsequently, they were contacted by phone and, if they were willing to participate and had a suitable mobile phone, they received an extensive information package. A total of 144 potential participants were invited, of whom 20 participated. Reasons for not participating were perceived burden due to participation in the study, lack of a suitable mobile phone, or inability to attend 1 of the 3 introduction meetings. The institutional review board of the Radboud University Medical Center approved the study, and participants signed an informed consent form at entry into the study (ID: 2015-2013).

### Study Procedures

We asked participants to record their blood glucose levels as usual and register hypoglycemic events in a personal diary in the 4 weeks prior to the start of the study. They were asked to keep a glucose (food and carbohydrate intake) and insulin dose diary for 5 days before starting to use the app. Participants visited 1 kickoff group meeting in which the app was installed and personalized by the nurse based on the diaries. Participants completed 3 validated questionnaires to assess diabetes-related emotional stress, fear of hypoglycemia, and diabetes self-care. After approximately 1 week of using the app, participants were contacted once for technical or medical support. In addition, medical support was available at all times for urgent matters, similar to regular care. After approximately 6 weeks of use, participants returned to the study center, where they repeated the questionnaires, including an additional survey about the usability of the app. Then, the app was removed from the device and respondents were interviewed individually. Before and at the end of the study period, we determined HbA_1c_.

### Measurements

We compared the hypoglycemic event rate during the 4 weeks before use of the app versus the hypoglycemic events recorded in the app and those logged by participants. The criterion for a hypoglycemic event was a measured blood glucose level below 4 mmol/L, with or without symptoms.

We measured diabetes-related emotional distress using the Problem Areas in Diabetes (PAID) scale, consisting of 20 items concerning negative emotions related to diabetes, resulting in a score from 0 to 100. The cutoff score for serious emotional distress is 40; average reported scores are 24.6 (SD 18.7) for type 1 diabetes [13].

The Hypoglycemia Fear Survey (HFS) [14] (in Dutch: *Angst voor Hypoglycemie Vragenlijst*) consists of 13 items, exploring worries and fears related to hypoglycemia. The sum of the scores is calculated, and higher scores indicate greater fear of hypoglycemia. The range is 0 to 52, and the cutoff score is 21 [13].

The Confidence in Diabetes Self-Care (CIDS) scale (in Dutch: *Diabetes zelfzorg vragenlijst*) [15] consists of 21 items and measures diabetes-specific self-efficacy—that is, the level of confidence that people with type 1 diabetes have to perform diabetes-specific self-care activities. It results in a score from 0 to 100, with higher scores indicating more trust. The questionnaire assesses trust or confidence in self-care, not whether the activities are actually done.

Participants completed the System Usability Scale (SUS) after the test period. The SUS provides a global view of subjective assessments of usability [16]. This short questionnaire consists of 10 items with 5 response options and results in a score from 0 to 100. The mean usability score for a system is 68; systems scoring 70 or above are considered to have acceptable usability, and those scoring above 72 are considered to have good usability [17].

### Semistructured Interviews

We held semistructured interviews at the end of the study to evaluate participants’ experiences with the app. These interviews focused on the advantages and disadvantages of using the app in daily practice, and on participants’ expectations for the future. An interview guide, based on guidelines for implementation and a framework for the evaluation of information systems in health care, was used [18,19]. This framework contains three domains: human, technology, and organization. In addition, we asked participants whether they would like to continue using this app and to rate the app on a scale from 1 (very poor) to 10 (excellent). All interviews lasted approximately 30 minutes and were performed face-to-face, recorded, and transcribed verbatim.

### Analysis

We performed analysis and statistics using IBM SPSS Statistics version 20 (IBM Corporation) and R version 3.2.0 (R Foundation). We did not calculate a formal sample size, given that we considered this to be an exploratory study; the aim was to include 20 participants in total. Normally distributed continuous variables were described as mean (SD). Median and interquartile values were determined when variables were not normally distributed. Qualitative or categorical variables (ie, baseline parameters) were described as frequencies and percentages. HbA_1c_ and survey scores for the PAID, HFS, and CIDS questionnaires before and after use of the app were compared by paired *t* tests. *P* values <.05 were regarded as statistically significant. We performed subgroup analyses based on activity: we calculated the number of median entries and created a least active group and a most active group, determined by the number of actions in the app.

We analyzed qualitative data using standard qualitative research methods. Two researchers independently analyzed the transcripts to identify barriers and facilitators that could affect use of the app, and perceived positive and negative effects of the app. They identified advice or suggestions for improving the next version of the app. All results were discussed until consensus was reached. Predefined tables were used to present results. Barriers and facilitators are presented following the framework of Gagnon et al [20]; positive and negative effects are presented according to Donabedian’s framework for quality of health care [21]. This framework distinguishes between process (eg, improved communication), structure (eg, hospital buildings), and outcomes (eg, death) of health care.

## Results

### General Results

In total, 20 people with diabetes were included, of whom 19 completed the study. We excluded 1 participant on the first day of the test, because of an ineligible mobile phone. Table 1 shows the participants’ characteristics. As intended, the study population was heterogeneous with respect to age, diabetes duration and glucose control, frequency of hypoglycemic events, and treatment: 12 people were on basal bolus, 7 on pump therapy, 3 used continuous glucose measurement, and 4 used a bolus calculator.

**Table 1:** General characteristics of the study population (n=19).

Characteristics		Values
Age (years), mean (SD)		43.8 (14.1)
**Sex, n (%)**		
	Male	7 (37)
	Female	12 (63)
Body mass index (kg/m^2^), mean (SD)		25.7 (3.4)
Duration of diabetes mellitus (years since diagnosis), mean (SD)		22.8 (14)
**Hemoglobin A_1c_**		
	%	7.9
	mmol/mol, mean (SD)	62.3 (7.8)
Insulin dose, U/day (range)		50 (11-100)
**Insulin regimen, n (%)**		
	Basal bolus	12 (63)
	Pump therapy	7 (37)

**Figure 1:**
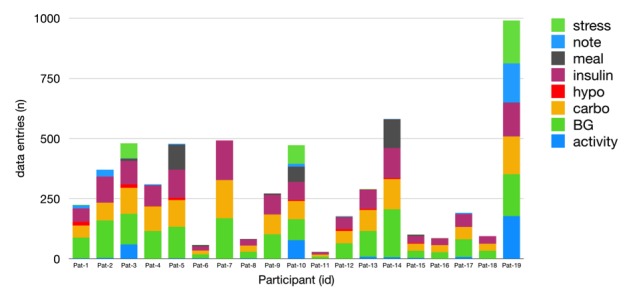
Use of app features by individual users. BG: blood glucose measurement; carbo: carbohydrate intake; hypo: hypoglycemic event.

### App Use

Over the study period (up to 6 weeks), a total of 5782 data entries were recorded, ranging from 29 data entries by the least active user to 990 data entries by the most active user (median 272). On average, participants recorded 6.8 logging entries during working days and 8.5 during weekend days. The proportion of active users decreased from 100% (19/19) in week 1 to 78% (15/19) after 4 weeks (Multimedia Appendix 2). The most frequently logged data were blood glucose (n=1740), insulin (n=1378), and carbohydrates (n=1366). Figure 1 presents participants’ use of the various app features.

### Pre- Versus Poststudy Comparison

Over the study period, mean HbA_1c_ dropped from 7.9% (62.3, SD 8 mmol/mol) to 7.6% (59.8, SD 7 mmol/mol; *P*=.047). The incidences of hypoglycemic events were 0.31 per participant per day at baseline and 0.27 per participant per day during the study period (*P*=.21). Basal to bolus ratio did not change over the study period.

Table 2 shows results of the questionnaires on disease burden. Mean diabetes-related emotional problems (PAID scale scores) decreased from 14.4 (SD 10.0) to 12.2 (SD 10.3; *P*=.04). Based on the dichotomized PAID score, 4 of the 19 respondents (21%) were at risk for emotional burnout (all scores ≥40), decreasing to 1 of 19 after the intervention period (5%). The score on the CIDS scale seemed to increase during the study period. The scores on the other PAID subscales, HFS, and CIDS scale did not change notably over the intervention period. The mean SUS score was 75.5 (SD 16.7, range 47.5-97.5) at the end of the study (n=19), indicating good usability. Multimedia Appendix 3 provides a comparison between more active and less active app users.

### Qualitative Results

Semistructured interviews led to several insights. All users rated the app, resulting in a mean score of 6.7 (on a scale from 1 to 10). A total of 8 respondents reported that they would prefer to continue to use the app if this were possible.

Frequently reported facilitators were the graphic display of blood glucose (trend) and ease of use of the app. However, a frequently mentioned barrier was also related to complexity of the app or that it was not easy to use. Another frequently mentioned barrier was that retrospective data entry was not possible in the app (although this was actually possible). Table 3 lists all reported facilitators and barriers for using the app.

Perceived positive and negative effects are presented according to the Donabedian framework for the quality of care. Among the six potential positive effects was that the app made participants more aware of their own situation and more conscious in managing their disease. The two negative effects that were mentioned were anxiety due to a bolus suggestion that did not reflect their personal view and the (risk of) more hypoglycemic events. Table 4 presents a complete overview of perceived positive and negative effects.

Other benefits that participants described from using the diabetes app were that it was a “wake-up call” and “refreshing,” since they had insufficient knowledge especially regarding carbohydrate counting. A total of 11 respondents indicated that they wished for a system with better (wireless) connections, such as a Bluetooth connection, between their blood glucose meter and the app, allowing for measurements to be imported, or even with a connection with their insulin pump. Independently of the app, 5 respondents also noted that they would like to have a continuous blood glucose sensor, allowing them to respond in a timelier manner. Regarding the possibility to share data from the app, 6 respondents mentioned that they would prefer better sharing options, such as easy exporting of data, use of cloud solutions, or a connection with their personal health record. The respondents would also have appreciated a more advanced way of presenting results in the app with graphs. Finally, respondents stated that the bolus suggestions could be more specific for different activities: 5 respondents mentioned that they were missing a sports mode function in the app.

**Table 2:** Results of Problem Areas in Diabetes (PAID), Hypoglycemia Fear Survey (HFS), and Confidence in Diabetes Self-Care (CIDS) questionnaires (n=19).

Instrument		Score, mean (SD)		*P* value
		Before	After	
**PAID scale**		20.0 (14.9)	17.2 (14.8)	.11
	Diabetes-related emotional problems	14.4 (10.0)	12.2 (10.3)	.04
	Treatment-related problems	2.1 (3.0)	1.3 (2.0)	.19
	Food-related problems	2.9 (2.6)	2.8 (3.0)	.82
	Social support-related problems	0.6 (1.0)	0.9 (1.4)	.24
HFS-Worry Scale^a^		25.4 (6.4)	25.3 (7.0)	.89
CIDS scale^b^		79.6 (11.3)	82.0 (10.9)	.13

^a^n=18, as 1 respondent did not answer item 12.

^b^n=17, as 1 respondent did not answer item 5 and 1 respondent did not answer item 10.

**Table 3:** Frequencies of barriers and facilitators for using the app.

App-related factors			Barrier	Facilitator
**Design and technical concerns**			**9**	**4**
	No internet access		2	0
	Adding medication: can add only half numbers or units (eg, 0.5)		1	1
	Retrospective data entry not possible		5	0
	Lack of notifications		1	0
	Graphic display of blood glucose (trend)		0	3
**Characteristics of the innovation**			**18**	**3**
	**Ease of use or complexity**			
		Meal picker complex or not intuitive	2	0
		Sliders too sensitive	2	0
		Complexity of app, easy to use (NFS^a^)	10	2
	**Relative advantage (usefulness) or lack**			
		No need to use additional booklet to register values	0	1
		NFS	4	0
**Validity of resources**			**9**	**2**
	Content available (completeness of meal picker)		3	1
	Frequency of advice (eg, lacking between 2 meals)		2	0
	**Bolus suggestion**			
		Does not take into account blood glucose trend	1	0
		Incorrect, does not correspond to personal view	3	0
		Also bolus suggestion, even when blood glucose is (too) low	0	1
**System reliability**			**3**	**0**
	Restarting the app takes too long		1	0
	Login issues		1	0
	Crashing (of app)		1	0

^a^NFS: not further specified.

**Table 4:** Frequencies of perceived positive and negative effects according to the Donabedian model for quality of care.

Outcome			Positive	Negative
**Processes**			**21**	**0**
	**Effects on psychological domains**			
		More aware or conscious of disease (self-)management	12	0
		Regularity: more frequent measurements	1	0
		More precise adjustments	1	0
		More frequent blood glucose checks	4	0
		Reduced number of corrections needed	1	0
	Patient education: better insulin advice, better than blood glucose meter		2	0
**Outcomes**			**18**	**3**
	Medication: reduced insulin use		1	0
	**Health status**			
		Weight loss	1	0
		More stable values (blood glucose, carbohydrates)	5	0
		Lower blood glucose levels, reduced number of high peaks	5	0
		Improved hemoglobin A_1c_	1	0
	Satisfaction: feeling more confident		3	0
	Knowledge: better knowledge about own glucose levels (graphs)		2	0
	Effects on psychological domains: anxiety, due to bolus suggestion that does not correspond to own estimation		0	1
	Health status: (risk of) more hypoglycemic events		0	2

### Bolus Suggestion

Logging and cloud storage allowed for subsequent analysis of several components of self-management. Of a total of 1378 insulin entries, 842 could be compared with the bolus calculator outputs. In 569 cases, the user accepted the bolus suggestion, whereas they reduced the suggested insulin dose in 101 cases and increased the insulin dose in 172 cases.

**Figure 2:**
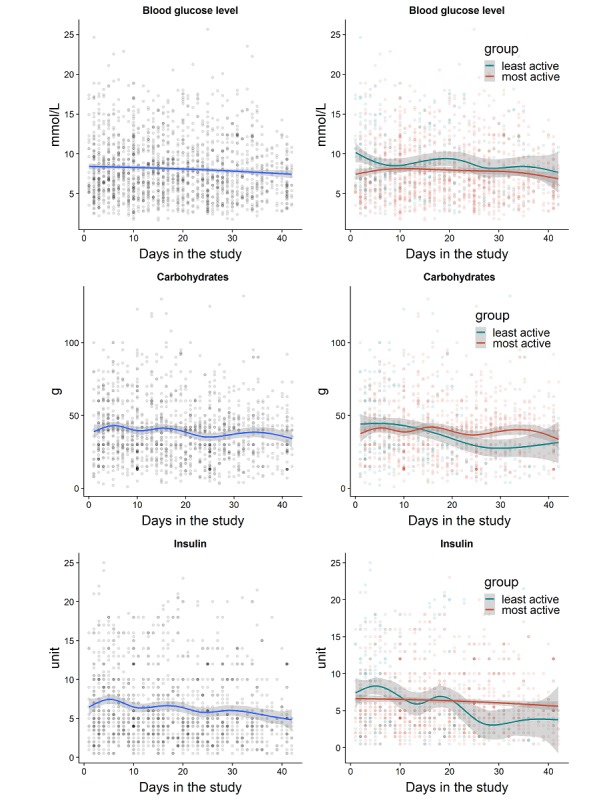
Blood glucose levels, carbohydrate intake, and insulin use per day in the study, for all participants (left) and stratified by user app activity: most and least active participants (right).

**Figure 3:**
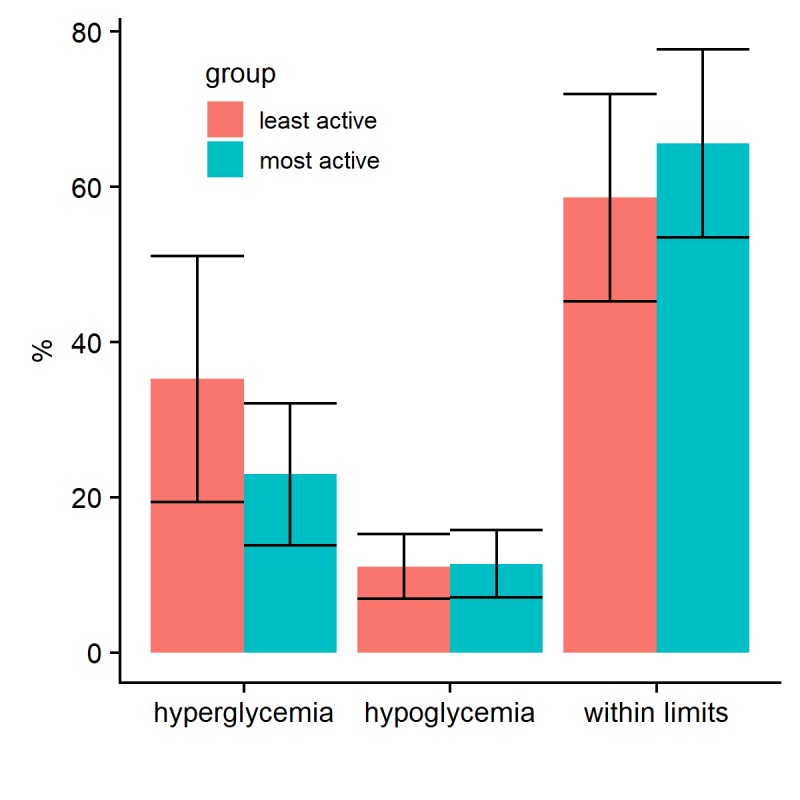
Percentage of blood glucose readings within normal limits, hyperglycemia, and hypoglycemia, stratified by user app activity: most and least active participants.

The logged dataset enabled us to compare glucose profiles after a bolus given according to the bolus suggestion versus boluses that were lower or higher than recommended, which were not different in this data set. More active users appeared to have more stable blood glucose levels, carbohydrate intake, and medication use (Figure 2). Compared with the least active participants, active participants tended to spend less time in hyperglycemia and more within the normal range (Figure 3). There were no differences in the drop in HbA_1c_ and disease burden between the least active and most active users.

## Discussion

### Principal Findings

This exploratory study with a prototype integrated mobile diabetes app in a heterogeneous sample of people with type 1 diabetes provided a detailed, in-depth, before-and-after analysis in an area with very limited evidence. The app, which uses the most relevant factors for diabetes self-management to provide a bolus suggestion, has the potential to benefit self-management, improve glucose control, and decrease disease burden. Logging and cloud storage allows for subsequent analysis of several components of self-management and potential feedback. The study revealed several barriers related to use of the app and identified high-priority areas for further development.

Over the short study period of 6 weeks, we noticed a significant improvement in glucose control with reduced hypoglycemia frequency and a significant decrease in disease burden. While this may have been an effect of using the app, the changes in glucose control could also be explained by a study effect. Participating in a study, keeping a diary, and discussing bolus settings increase the time and attention people with diabetes devote to their treatment. Measurement of disease burden did confirm the high burden associated with diabetes. While the decrease in disease burden may also have been a study effect, it has also been reported that PAID scores do not easily change over time [22]. Even when the changes are caused by a study effect, use of the app can apparently catalyze more attention toward diabetes management, without increasing disease burden.

The app combines several features, many of which are found in other diabetes devices, such as insulin pumps and glucose meters, or offered as stand-alone functions in mobile apps. Still, to our knowledge, the combination of most functions that are considered basic for self-management, including blood glucose logging, insulin dosage, carbohydrate measurement, exercise, graphics, and chat and direct contact with health care providers on a mobile phone app, is unique to this app [9]. The participants were generally positive about the app, with most (79%) still actively using it after 4 weeks, and a large proportion stating that they would prefer to continue using it after the study. More specifically, the bolus calculator was evaluated as a relevant feature, and the app made them more aware of their diabetes self-management. These results are in line with findings of a qualitative study among adults with type 1 diabetes, which found that users of an app with bolus suggestion generally trusted the suggestion [23]. The bolus suggestion function was new to most study participants, while 2 participants already using a bolus calculator also reported that their personal settings as added by the nurse needed to be updated. This illustrates one of the problems of the bolus calculator: optimal use requires, first, an appropriate determination of the insulin to carbohydrate ratio and a correction factor and, subsequently, frequent and repeated fine-tuning of the settings. While the use of a bolus calculator has been associated with a slight improvement in glucose control [24], particularly among pump users [25], not all authors have identified benefits [26], and patients not on a pump rarely use a bolus calculator. In another study, our own group found improvements in neither glucose control nor disease burden after a structured introduction of a bolus calculator to experienced pump users versus carbohydrate and ratio education alone [27]. In this study, the bolus suggestion seemed to provide an educational element in reinforcing the relationship between insulin use and carbohydrate intake.

Our study identified several educational gaps among the participants, particularly at the level of carbohydrate counting. While all participants had followed a structured diabetes education, including dietary aspects and carbohydrate counting, which had generally been repeated over time, detailed discussion of diaries unmasked a lack of knowledge or wrong understanding. This is not unusual among people with long-standing diabetes, and particularly detailed carbohydrate counting is challenging and requires a substantial time investment. For some, this may be more than they can or are willing to invest in the disease management. While more intense and repeated education may be required, use of an integrated app preferably with detailed feedback may present an opportunity to provide tailored education.

### Other Studies

Given the dearth of available apps for diabetes management, the lack of supporting scientific evidence is compelling. Appropriate studies on relevant outcome parameters are scarce, particularly in type 1 diabetes. In addition, most studies have focused on improvements in glucose control (HbA_1c_). Our primary aim was to support people with diabetes in proper decision making, hopefully resulting in decreased disease burden. A recent review by Hood et al of studies that reviewed apps, both controlled and uncontrolled [10], identified that several studies in which HbA_1c_ was significantly reduced were of poor quality. Brzan et al reviewed 9 of approximately 500 diabetes apps available in the Apple App Store, and identified 1 app containing a bolus calculator that had been shown to prevent hypoglycemic events [9]. A meta-analysis [12] summarizing controlled app studies identified 3 apps for type 1 diabetes, with 2 having no effect and 1 having a nonsignificant effect on HbA_1c_. Effects on other parameters were not studied. Logging and cloud storage of data allows for subsequent analysis of several components of self-management and potential feedback. In our study, we analyzed the use of the bolus suggestion, the glucose pattern, insulin use, and carbohydrate intake before and after a hypoglycemic event and the differences between most frequent and least frequent users. Given the relatively small sample size and short study duration, the additional analyses allowed for only limited conclusions. Nevertheless, we think this possibility has great potential in identifying individual profiles, particularly when combined with personalized feedback. While more authors are identifying these potentials, this area is still in its infancy. More robust study designs including a control group are needed to formally assess the effects of self-monitoring apps.

Our study also identified several barriers to using the app, most of which were related to usability issues, such as lack of direct connectivity with devices. Another known barrier is the time needed to add information about nutrition and insulin [9]. Although we did not calculate the specific time needed, our qualitative analysis revealed that users found it time consuming, which could negatively influence app use. Future development should aim for automatic connections; this seems to be feasible with measuring devices (see below) and is already customary with pumps. Recent developments of Bluetooth-connected insulin pens [28] may further complement automatic input. Finally, optical recognition of numbers on glucose meters does not work properly under circumstances of decreased light. It should also be realized that in current practice the choice of a given glucose meter heavily depends on reimbursement issues. While input through a ruler was rapid and convenient, direct connection would still be optimal. The emergence of continuous glucose sensors that can directly connect to mobile apps may help in overcoming these barriers.

### Strengths and Limitations

Our study had several limitations that are related to the exploratory design. These include the small sample size, open uncontrolled study design, and relatively short duration of follow-up. Obviously, mobile phone use and brand version determine patient selection. Intentionally, we did not select study participants based on treatment (pump or multiple daily injections) or technical savvy. Strengths of the study are that the app contains all of the basic features for optimal self-management and our use of the mixed-methods approach, which allowed for both comparison of objective measures before and after the study and assessment of subjective user experiences. Furthermore, the large (logged) complete data set allowed for a wealth of valuable analyses.

### Conclusion

This study suggests that an integrated mobile phone app has the potential to benefit self-management, improve glucose control, and decrease disease burden. It may help to better integrate glucose measurements, carbohydrate intake, physical activity, and insulin dose and can identify educational gaps. Logging and cloud storage allows for subsequent analysis of several components of self-management and potential feedback. Finally, the study revealed several barriers to the use of the app and identified high-priority areas for further development. Clearly, further work is needed to advance digital support for people with type 1 diabetes.

## Appendix

Multimedia Appendix 1 App screenshots.

Multimedia Appendix 2 App use (6 weeks).

Multimedia Appendix 3 Least and most active app users.

## Abbreviations

CIDS: Confidence in Diabetes Self-Care
HbA_1c_: hemoglobin_1c_
HFS: Hypoglycemia Fear Survey
PAID: Problem Areas in Diabetes
Radboudumc: Radboud University Medical Center
SUS: System Usability Scale
