# Supplementary figures and images for: Glucose Control, Disease Burden, and Educational Gaps in People With Type 1 Diabetes: Exploratory Study of an Integrated Mobile Diabetes App
Source: JMIR Diabetes. 2018 Nov 23;3(4):e17. doi: 10.2196/diabetes.9531 (PMC6286423; doi:10.2196/diabetes.9531)

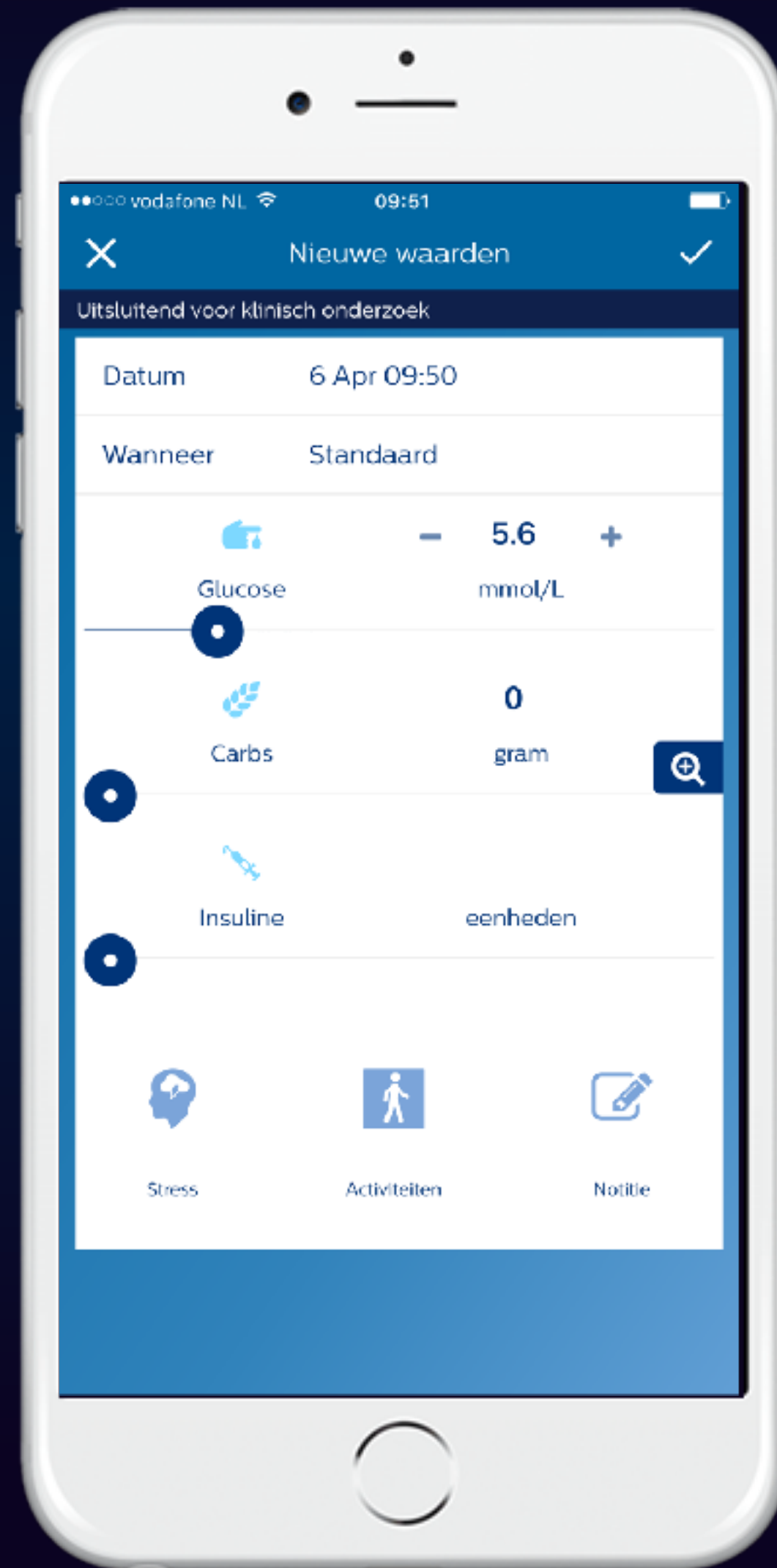

Adding user's data\*

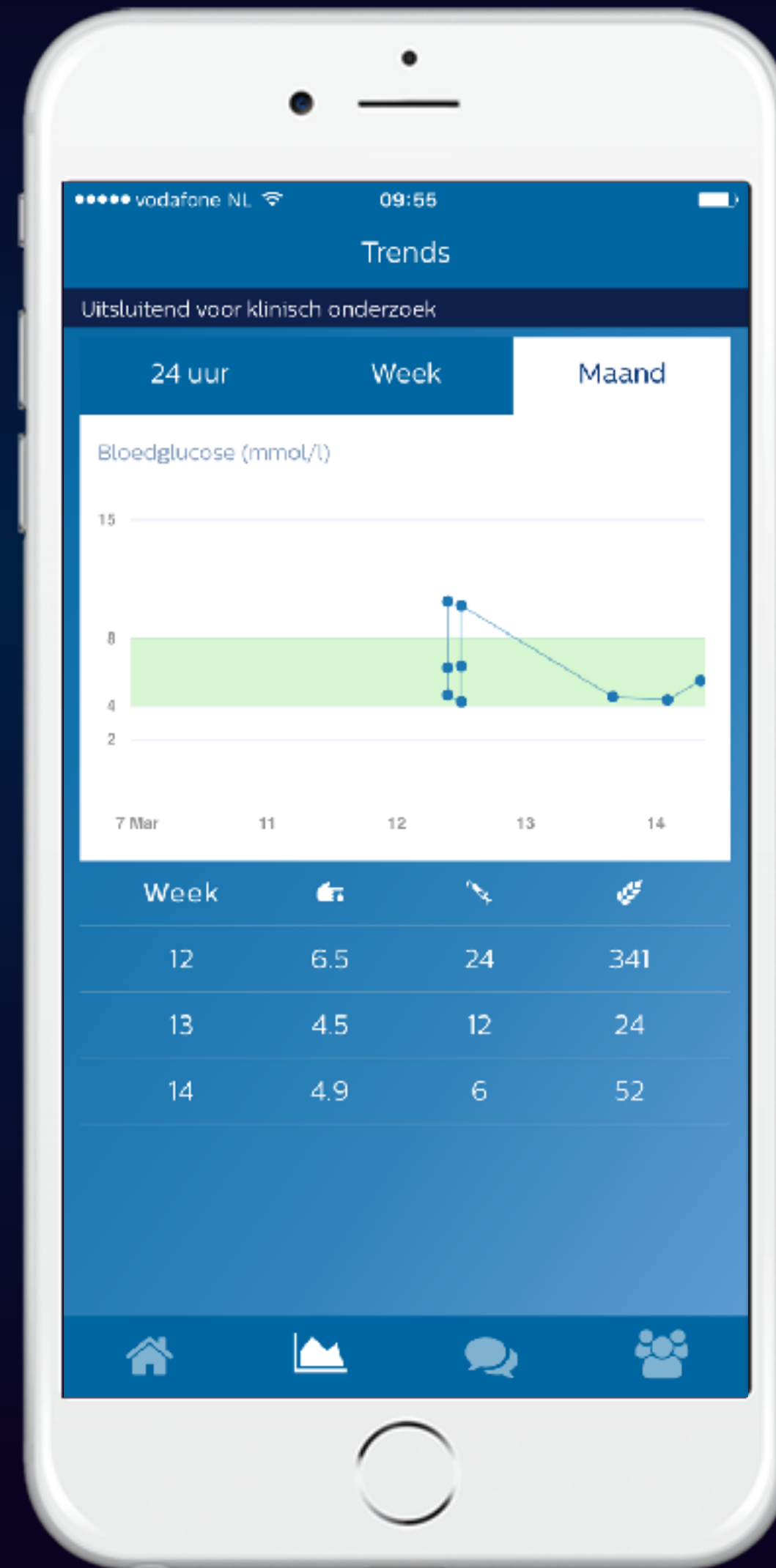

Trends\*

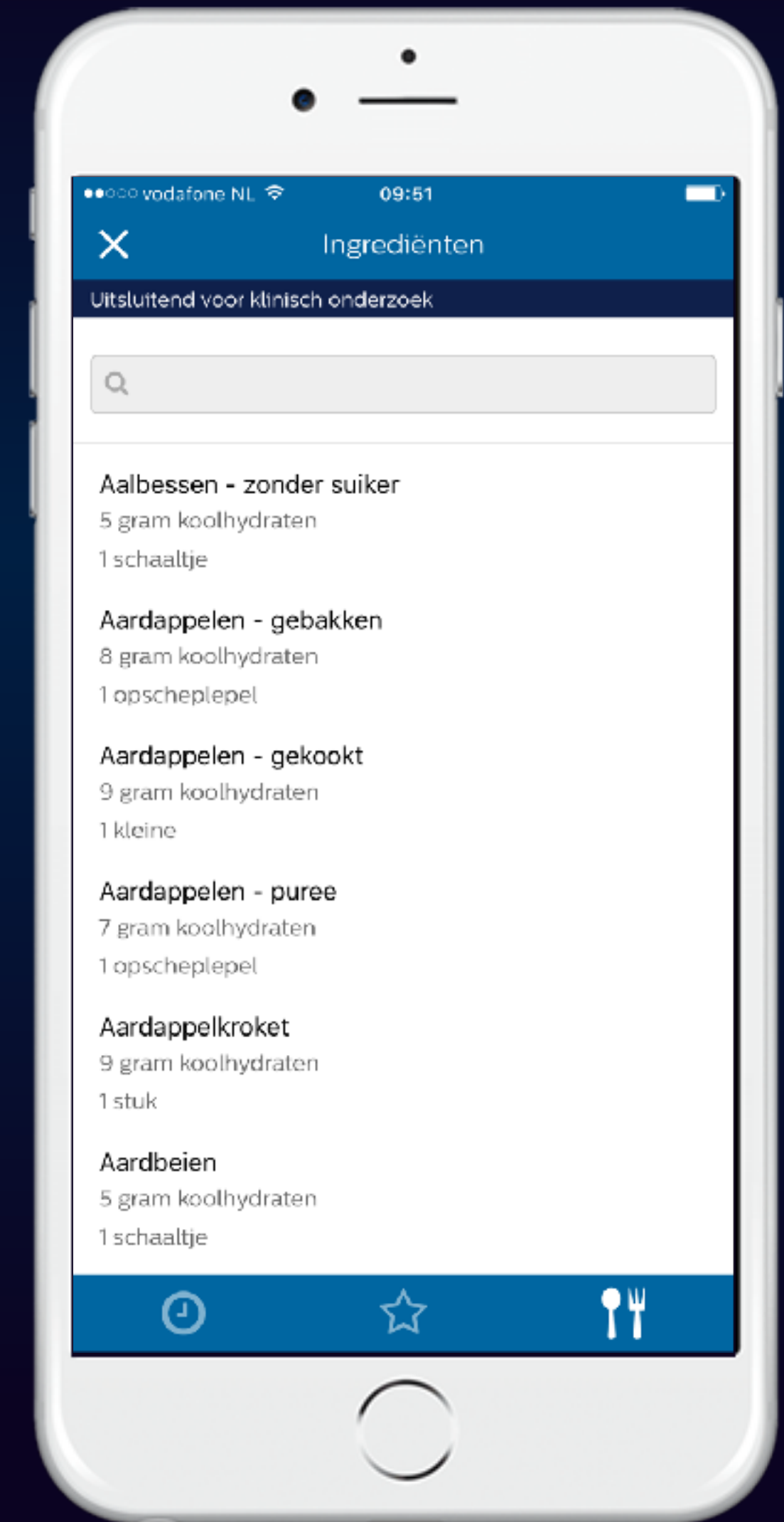

Meal picker\*

Supplement: Multimedia Appendix 1 [file diabetes_v3i4e17_app1.pdf]

## Supplementary file 1: App usage (six weeks).

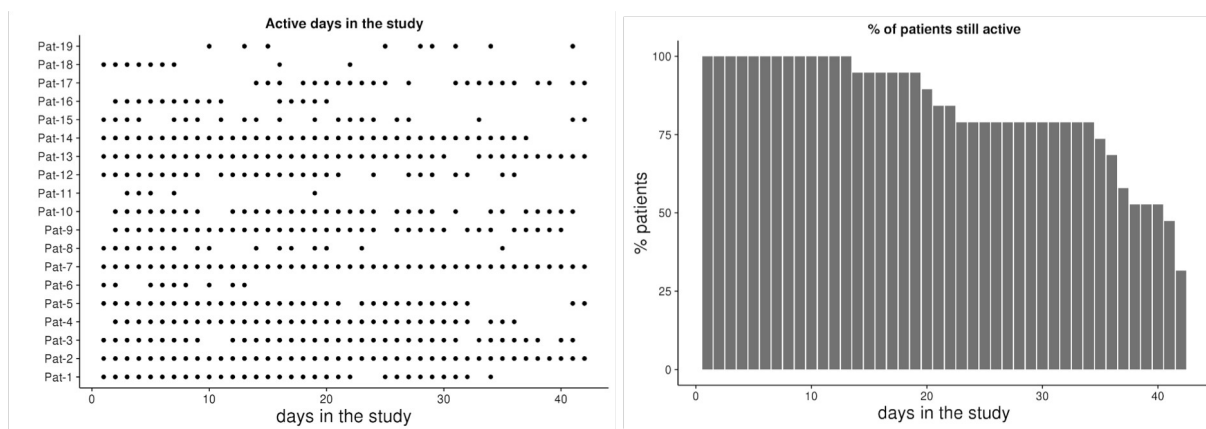

Supplement: Multimedia Appendix 2 [file diabetes_v3i4e17_app2.pdf]
