# Supplementary material for: Glucose Control, Disease Burden, and Educational Gaps in People With Type 1 Diabetes: Exploratory Study of an Integrated Mobile Diabetes App
Source: JMIR Diabetes. 2018 Nov 23;3(4):e17. doi: 10.2196/diabetes.9531 (PMC6286423; doi:10.2196/diabetes.9531)
Supplement: Multimedia Appendix 3 [file diabetes_v3i4e17_app3.pdf]

Supplementary file 2: Least and most active patients

			Least active	Most active
Demographics		AGE	37.67	49.40
		Gender	6 Female, 3 Male	6 Female, 4 Male
Clinical		BMI	25.50	25.82
		DM.Years	23.44	22.30
		HbA1C.before.study	63.33	61.30
		HbA1C.after.study	62.00	57.80
PAID - Problem Areas in Diabetes	Total	before	27.64	13.13*
		after	24.72	10.38*
		difference	-2.92	-2.75
	Emotional	before	19.86	9.50*
		after	16.81	8.13
		difference	-3.06	-1.38
	Trx	before	2.22	2.00
		after	2.08	0.50
		difference	-0.14	-1.50
	Food	before	4.72	1.25**
		after	4.44	1.25*
		difference	-0.28	0.00
	Social	before	0.83	0.38
		after	1.39	0.50
		difference	0.56	0.13
HFS - Hypoglycemia Fear Survey		before	28.11	23.50
		after	27.13	23.90
		difference	-0.75	0.40
CIDS - Confidence in Diabetes Self-Care Scale		before	77.81	81.25
		after	79.58	84.63
		difference	2.34	2.36
